# Allocation of Users of Mental Health Services to Needs-Based Care Clusters: An Italian Pilot Study

**DOI:** 10.1007/s10597-023-01200-3

**Published:** 2023-10-26

**Authors:** Angelo Barbato, Barbara D’Avanzo, Giovanni Corrao, Teresa Di Fiandra, Lucia Ferrara, Andrea Gaddini, Carlotta Micaela Jarach, Matteo Monzio Compagnoni, Alessio Saponaro, Salvatore Scondotto, Valeria D Tozzi, Antonio Lora

**Affiliations:** 1 Laboratory of Quality Assessment of Care and Services, Department of Health Policy, Istituto di Ricerche Farmacologiche Mario Negri IRCCS, Milan, Italy; 2 Unit of Biostatistics, Epidemiology and Public Health, Department of Statistics and Quantitative Methods, University of Milano-Bicocca, Street Bicocca degli Arcimboldi, 8, Building U7, 20126 Milan, Italy; 3 National Centre for Healthcare Research and Pharmacoepidemiology, University of Milano-Bicocca, Milan, Italy; 4 Psychologist, previously General Directorate for Health Prevention, Italian Ministry of Health, Rome, Italy; 5 Centre of Research on Health and Social Care Management, CERGAS SDA Bocconi School of Management (Bocconi University), Milan, Italy; 6 Agency for Public Health, Lazio Region, Rome, Italy; 7 Laboratory of Lifestyle Epidemiology, Department of Environment and Health, Istituto di Ricerche Farmacologiche Mario Negri IRCCS, Milan, Italy; 8 General Directorate of Health and Social Policies, Emilia-Romagna Region, Bologna, Italy; 9 Department of Health Services and Epidemiological Observatory, Regional Health Authority, Sicily Region, Palermo, Italy; 10 Department of Mental Health and Addiction Services, ASST Lecco, Lecco, Italy

**Keywords:** Epidemiology, Determinants of Health, Prevention, Public Mental Health, MHCT, Public Health

## Abstract

In Italy, despite strong community-based mental health services, needs assessment is unsatisfactory. Using the Mental Health Clustering Tool (MHCT) we adopted a multidimensional and non-diagnosis dependent approach to assign mental health services users with similar needs to groups corresponding to resources required for effective care. We tested the MHCT in nine Departments of Mental Health in four Italian regions. After a brief training, 318 professionals assessed 12,938 cases with a diagnosis of schizophrenia, depression, bipolar disorder and personality disorder through the MHCT. 53% of cases were 40–59 years, half were females, 51% had a diagnosis of schizophrenia, 48% of cases were clinically severe. Clusters included different levels of clinical severity and diagnostic groups. The largest cluster was 11 (ongoing recurrent psychosis), with 18.9% of the sample, followed by cluster 3 (non-psychotic disorders of moderate severity). The MHCT could capture a variety of problems of people with mental disorders beyond the traditional psychiatric assessment, therefore depicting service population from a different standpoint. Following a brief training, MHCT assessment proved to be feasible. The automatic allocation of cases made the attribution to clusters easy and acceptable by professionals. To what extent clustering provide a sound base for care planning will be the matter of further research.

## Introduction

The rise of a community-based model of mental health care all over the world in the last years led to an increasing focus on care planning and coordination based on a patient-centered approach, requiring a careful assessment of multiple health and social needs of people experiencing mental disorders. However, although a number of needs assessment tools have been developed (Thornicroft et al., 2016), assessment and classification of people with mental disorders in everyday practice of mental health services is mainly based on psychiatric diagnosis, whereas use of need-led instruments is restricted to research purposes (Boswell et al., 2015). However, the utility of psychiatric diagnosis as a starting point for treatment planning and identification of resources for effective care is being increasingly questioned, despite the ongoing refinements of the diagnostic systems (Maj, 2020). Heterogeneity in psychiatric diagnostic classification (Allsopp et al., 2019), blurred boundaries between categories (Parnas, 2015) and inflated comorbidity (Van Loo & Romeijn, 2015) are common problems in clinical practice. Moreover, needs are closely related to disability as well. A recent worldwide survey showed that most clinicians find ICD-10 or DSM-5 labels suitable for administrative purposes only (First et al., 2018). Needs assessment based on diagnosis remains a challenge for community-based services.

The Research Domains Project aimed to develop a new classification cutting across the boundaries of current diagnoses relying on advances in genetics and neurosciences (Cuthbert & Insel, 2013). However, this approach is unlikely to provide a sound foundation for mental health care, unless reliable biomarkers for mental disorders are identified, which is far from being achieved (Carvalho et al., 2020).

To circumvent the drawbacks of a diagnosis, allowing the introduction of a standardized way to assess needs and predict resources utilization in routine psychiatric services, the so-called mental health clustering, was introduced in the United Kingdom about ten years ago (Self et al., 2007). Mental health clustering is based on a multidimensional approach, independent of the diagnosis, to assign people who present similar characteristics and needs to homogeneous groups. Allocation of a single service user to a care cluster is the result of a comprehensive problem-oriented assessment, including mental and physical health, social functioning, interpersonal and environmental context, self-perception of problems and relation with services. This should predict the resources required for effective care and provide the background for innovative models of funding mental services in relation to packages of care, moving away from fee-for-service or block contract financing (Jacobs, 2014).

Cluster allocation is performed by a trained mental health professional or by a professional team through the Mental Health Clustering Tool (MHCT), which is used to assign service users to one of 21 care clusters (NHS England 2019). Table 1 provides a brief description of the clusters and shows the rank of each cluster according to the level of complexity and needs, calculated of the basis of the resources allocated by the UK mental health system for care provision (Moscelli et al., 2019). Cluster 9 is left blank for pending assessment.

**Table 1:** Definitions of the clusters of the Mental Health Clustering Tool. QUADIM project, Italy, 2015–2016

Cluster	Definition	Rank
1	Common mental health problems of a low severity	**19**
2	Common mental health problems of a low severity but greater need	**18**
3	Non-psychotic disorders of a moderate severity	**17**
4	Severe non-psychotic disorders	**15**
5	Very severe non-psychotic disorders	**12**
6	Non-psychotic disorders of over-valued ideas	**14**
7	Enduring non-psychotic disorders (high disability)	**11**
8	Non-psychotic chaotic and challenging disorders	**9**
10	First episode of psychosis	**6**
11	Ongoing recurrent psychosis of low symptomatology	**13**
12	Ongoing recurrent psychosis with high disability	**8**
13	Ongoing recurrent psychosis of high symptomatology and high disability	**3**
14	Psychotic crisis	**2**
15	Severe psychotic depression	**4**
16	Psychosis and affective disorder (high substance misuse and engagement)	**5**
17	Psychosis and affective disorder difficult to engage	**1**
18	Cognitive impairment (low need)	**20**
19	Cognitive impairment or dementia complicated (moderate need)	**16**
20	Cognitive impairment or dementia complicated (high need)	**10**
21	Cognitive impairment or dementia (high physical or engagement needs)	**7**

Ranking from 1 (Highest complexity/need) to 20 (Lowest complexity/need)

The MHCT is a clinician-rated instrument, including the 12 items of the Health of the Nation Outcomes Scales (HoNOS) (Wing et al., 1998), plus an additional item and the five items of the Summary Assessment of Risk and Need (SARN) (Self et al., 2007). It is used to assess needs on a current and historical basis. The time frame for assessment is two weeks prior to rating date for eleven HoNOS items. For other items, scores are attributed on the base of people’s usual situation.

**Fig. 1:**
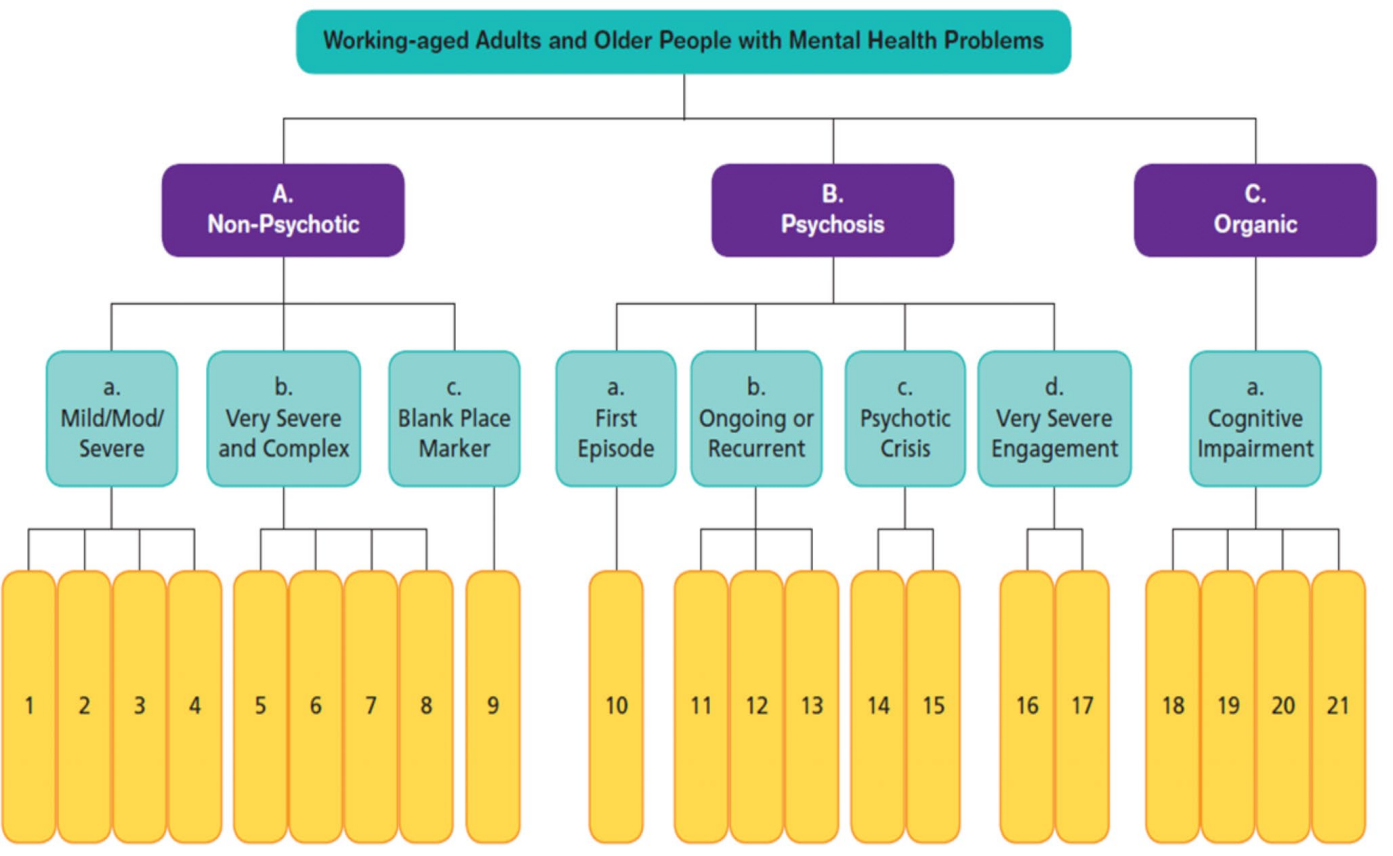
Decision tree for cluster allocation

Each item is rated by staff on a scale from 0, “no problems”, to 4, “severe to very severe problems”, considering all the available information on patients’ situation. According to a guidance, raters translate the scores into assignments to the 21 clusters, reflecting an integrated evaluation of clinical severity, functional impairment, treatment needs, social context and relation with services (Mental Health Clustering Booklet, version 5.0, 2016/2017, NHS England Publications Gateway Reference 04421). The decision tree leading to the allocation to clusters is shown in Fig. 1.

First, the raters assign a case to one of three so-called super classes; second, they proceed to identify the most appropriate cluster included in the selected super class. Anyway, the clinicians are free to override the guidance indications and assign the patient to a different cluster according to their own judgment. The final assignment to clusters can also be made through a computerized algorithm, which assigns the patient to the cluster that fits his/her scores with the highest probability. After a period of time specific for each cluster, or sooner if patient status changes significantly, a review of the cluster allocation needs to be performed.

Although clustering is now mandatory in adult mental health services in United Kingdom, its implementation has been slow and the debate on its impact on quality and outcome of care is not settled (Twomey et al., 2015). Some criticisms about the clustering process have been raised mainly in relation to two aspects: the burden for the clinicians and the possible bias introduced in the case assessment by the link between the cluster definition and the allocation of resources to services. This could imply a shifting for clinicians from a concern in providing the best service to a concern in getting higher financial incentives (Yeomans, 2014). The perverse behaviors of organizations and individuals trying to game the payment systems, through upcoding, creaming or dumping have been well documented in hospital medical care (Cots et al., 2011). It is also worth noting that clustering can be used as a framework for two different approaches to funding of mental health care, namely episodic and capitation payment. Evidence about which payment model is more suitable to enhance services results in meeting patient needs is still lacking (Jacobs et al., 2018).

Overall, outcome research on implementation of mental health clustering is limited and no data are available on its use in mental health services outside the United Kingdom. To move a step towards filling this gap, this paper will present and discuss a pilot project aimed at testing the feasibility of the utilization of the MHCT in a large sample of Italian psychiatric services, describing the results of the allocation of service users to needs-based clusters, verifying to what extent the MHCT can provide a needs evaluation overcoming the limitations of the customary psychiatric assessment.

We decided to conduct this study because of the consideration that needs assessment is unsatisfactory in Italy, despite its community-based mental health system (Galderisi et al., 2020).

## Methods

This study was a component of a project sponsored by the Italian Ministry of Health and the National Center for Disease Control and Monitoring, using data from the National Mental Health Information System to link service use, consumption patterns, costs, and socio-demographic characteristics of users in Italy with needs-based clusters. The overall aim was to verify to what extent consumption patterns and costs could be predicted by the cluster allocation.

This was an observational study carried out in nine Departments of Mental Health (DMH) of four Italian areas (the entire regions of Lombardy, Emilia-Romagna, Lazio and the province of Palermo in Sicily). In Italy, the DMH is the National Health Service agency providing mental health care to the population of a defined catchment area through a network of community services. The organisational model of DMHs is based on multi-disciplinary teams, including psychiatrists, psychologists, nurses, social workers, occupational therapists, rehabilitation counsellors, auxiliary staff and in some cases peer support workers. Each DMH should be able to provide the full range of psychiatric care, from acute emergency treatment to long-term rehabilitation, therefore it should include one or more of the following services: community mental health centers, outreach teams, general hospital inpatient units, day care centers, community residential facilities and supported housing programs (Barbui et al., 2018). Community mental health centers are the entry point to the system, with the exception of emergency hospital admissions. The catchment areas of the nine departments had a total population of 5,3 million, namely 10.5% of the adult country residents. A total of 53 community mental health centers were identified as the settings for the administration of the MHCT to all consecutive cases with a diagnosis of severe mental disorders attending the service over three months. A project coordinator was appointed in each center. For the scope of this study, the definition of severe mental illness covered four diagnostic groups: schizophrenia spectrum disorders (ICD-9 codes 295, 297, 298; ICD-10 codes F20-F29); major depression (ICD-9 codes 296, 296.2, 296.3, 296.9; ICD-10 codes F32-F39); bipolar disorders (ICD-9 codes 296.0–1; 296.4-8; ICD-10 codes F30-F31) and personality disorders (ICD-9 -codes 301; ICD-10 codes F60-F69). It is worth noting that in three regions (Emilia-Romagna, Lazio, Sicily) the ICD-9 system is used and in one region (Lombardy) the ICD-10. The diagnosis registered in the regional information systems was considered. No attempt was made to review the diagnostic assessment.

The study comprised the following phases: (1) preparation of the Italian version of the MHCT and the related instructions for use, (2) training of mental health professionals of participating services in the administration of the MHCT, (3) data collection and cluster allocation, (4) data analysis.

For the translation of the MHCT, the previously developed and assessed for reliability Italian version of the HoNOS was used (Lora et al., 2001). The MHCT training in the methodology of MHCT use encompassed the use of the version 5.0 of the Mental Health Clustering Booklet. Between October and December 2017, a total of 447 professionals were trained to use the MHCT in two-day sessions by two of the authors. The training sessions took place in the centers participating in the project. Where the number of participating professionals was more than twenty, more sessions were held. Training involved 218 medical doctors, 152 nurses, 39 psychologists, 28 occupational therapists/educators, 7 social workers, and 3 other professionals.

After the training, the professionals were asked to administer the MHCT to all patients they entered in contact with between January and April 2018 and score the individuals’ needs according to the guidance of the Mental Health Clustering Booklet. The MHCT scores were introduced in a platform ad hoc developed. The platform allowed the project coordinators of centers to monitor the records of all the professionals of that area, whereas raters could just access their own records. Corrections of data in the platform could be made by the coordinators only. We checked for data quality considering completeness and consistency of data in each record and asked the coordinator to look for clarification and completion when necessary. Once MHCT administration and data check were completed, the ratings of each MHCT record were allocated to a super class through a computerized algorithm designed by the project research group in accordance with the methodology derived from the Mental Health Clustering Booklet guidance. Following the allocation to the super classes, each case was assigned to the best fit cluster, using the version 2.5 of the Technical Guidance for the MHCT assessment algorithm.

(https://assets.publishing.service.gov.uk/government/uploads/system/uploads/attachment_data/file/214910/Mental-Health-clustering-support-tool-algorithm.pdf).

Considering the above-mentioned criticisms of the mental health clustering procedures, we decided to modify the protocol used in the United Kingdom, with the aim of easing the clinicians’ burden and limiting the possibility of discretionary coding. Therefore, overriding the algorithm indications by the clinicians was not allowed.

Data analysis was conducted descriptively. Patients were described by means of figures and proportions in categories of age, gender, diagnosis, center, cluster, degree of probability in cluster attribution and clinical severity according to the HoNOS score. As proposed by Lelliott (1999), patients scoring > 2 in at least one item were considered as clinically severe. We cross-tabulated clusters according to gender, age class, diagnosis, center and degree of probability of cluster allocation. Associations between the variables were tested by means of Pearson’s chi-squared test. Analyses were conducted using JMP Pro 15, SAS Institute Inc.

## Results

Training was assessed as satisfactory or very satisfactory by 83% of the trainees. Self-reported burden associated with the clustering process was considered as low by most trainees. A total of 318 professionals (71% of those trained) assessed 13,291 patients. Data quality check identified 353 (3%) incorrect or incomplete MHCT score sheets. Therefore, the final database consisted of 12,938 cases. 6,484 records concerned women and 6,554 men; 3,274 people were aged 40–49 years and 3,618 were 50–59, accounting for 53.3% of the whole sample. Most of the sample had a diagnosis of schizophrenia and related disorders, and the smallest diagnostic group was bipolar disorder, with 1,776 cases (13.7%). According to the HoNOS, 6,167 (47.7%) cases were clinically severe and 6,771 (52.3%) non-severe. The sample characteristics are summarized in Table 2.

**Table 2:** Characteristics of 12,938 patients assessed. QUADIM project, Italy, 2015–2016

	N	%
Sex		
Female	6,484	50.1
Male	6,454	49.9
**Age**		
≤ 29	1,149	8.9
30–39	1,668	12.9
40–49	3,274	25.3
50–59	3,618	28.0
60–69	2,123	16.4
≥ 70	1,106	8.5
**Diagnosis**		
Schizophrenia	6,584	51.0
Depression	2,166	16.7
Bipolar disorder	1,958	15.1
Personality disorder	2,230	17.2

Table 3 presents the results of the allocation to clusters: the cluster including more cases was 11 (ongoing recurrent psychosis), with 2,447 cases (18.9% of the sample), followed by cluster 3 (non-psychotic of moderate severity) with 2,289 cases (17.6%), cluster 12 (ongoing recurrent psychosis with high disability) with 1,328 cases (10.3%), and cluster 18 (cognitive impairment of low need) with 1,290 cases (10%). The clusters including less cases were clusters 10, 6 and 15, with less than 100 cases, followed by cluster 5 with 220 cases (1.7%) and cluster 2 with 380 cases (2.9%).

**Table 3:** Attribution of 12,918 patients to the clusters. QUADIM project, Italy, 2015–2016

Cluster	N	%
1	887	6.9
2	380	2.9
3	2,280	17.6
4	846	6.5
5	220	1.7
6	44	0.3
7	539	4.2
8	189	1.5
10	19	0.1
11	2,447	18.9
12	1,328	10.3
13	560	4.3
14	295	2.3
15	87	0.7
16	83	6.4
17	322	2.5
18	1,290	10.0
19	536	4.1
20	193	1.5
21	373	2.9

Considering the complexity of clusters, as defined by the level of resources needed for an effective care (Moscelli et al., 2019), three out of four among the most frequent clusters ranked low in terms of complexity and needs for care. The opposite held true for two out of four clusters among the less frequent ones.

Allocation to clusters differed across regions. In all regions cluster 11 was the most prevalent, but in Lombardy it was 2.2-fold more frequent than in Lazio with 864 (24% in the region) and 194 cases (11% in the region) respectively. In all regions except in Lombardy cluster 3 was the most prevalent (data not shown).

**Fig. 2:**
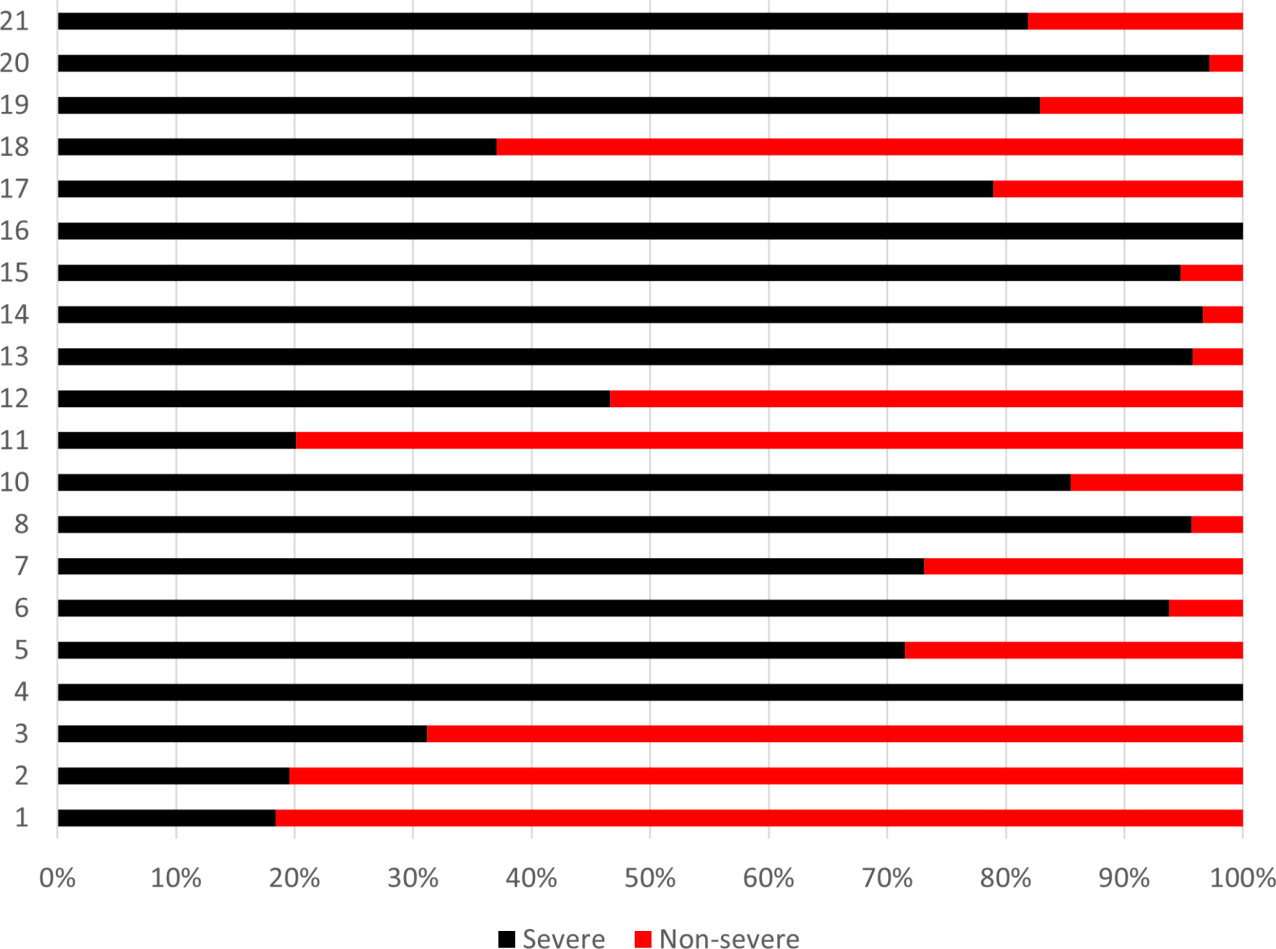
Distribution of 2,918 cases according to clinical severity across clusters

Figure 2 shows that the clusters were characterized by different levels of clinical severity. Clusters 4 and 16 had only severe cases, and nine clusters had more than 50% of severe cases. Non-severe cases were more than 50% in clusters 1, 2, 3, 11, 12 and 18.

**Fig. 3:**
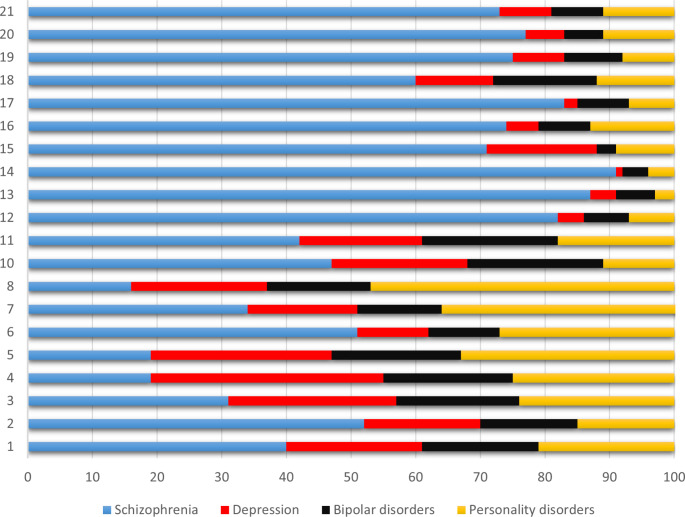
Distribution of cases according to diagnosis across clusters

As shown in Fig. 3, all diagnostic groups were represented in all clusters. Cases with a diagnosis of schizophrenia were at least 50% in all clusters except 1, 3, 4, 5, 7, 8, 10, 11.

**Fig. 4:**
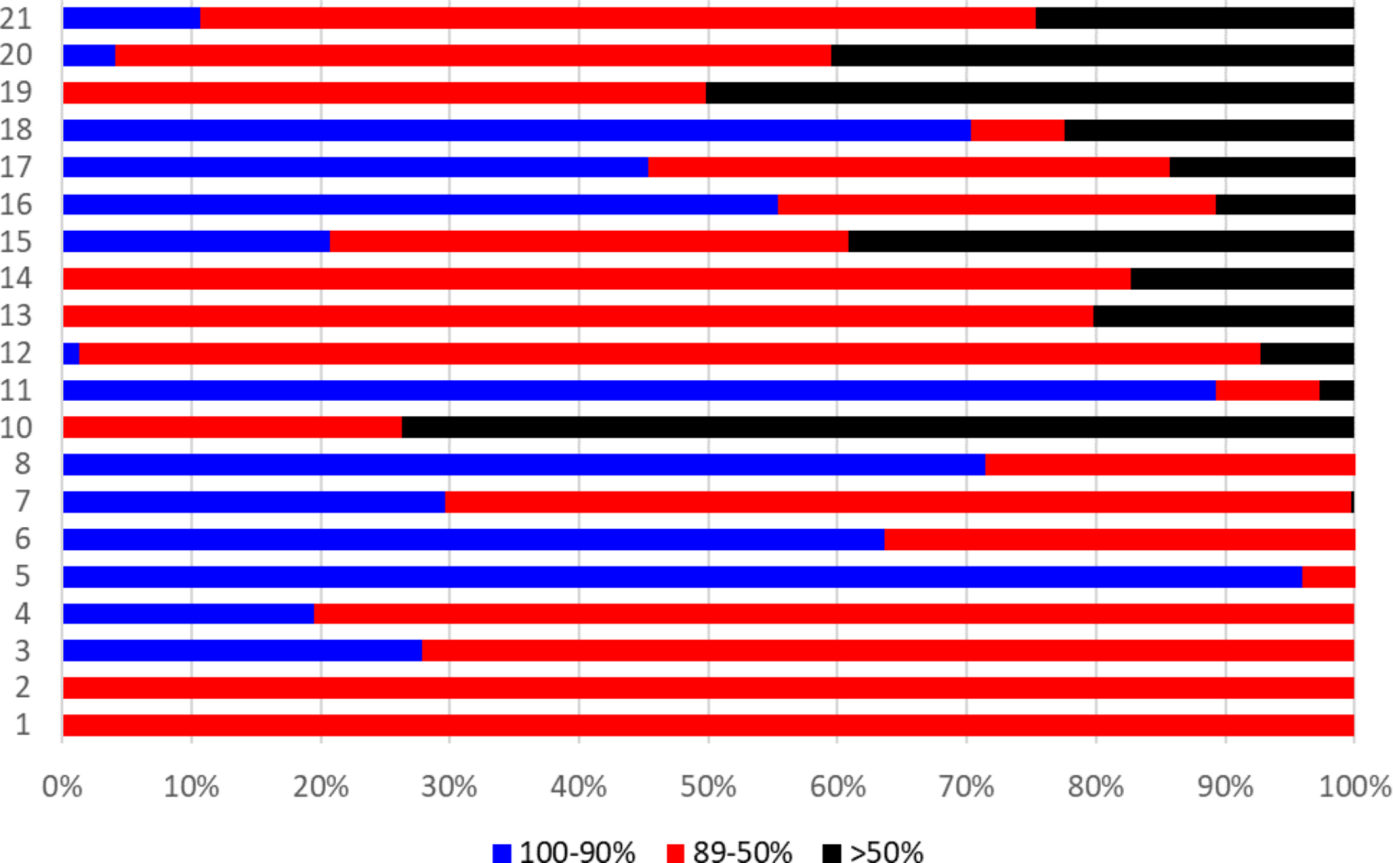
Distribution of cases according to the degree of probability of the allocation to clusters

Figure 4 shows that the degree of probability in the allocation of cases to the best fit cluster varied greatly across clusters. Clusters with more than 50% of cases allocated with a very high probability were 5, 11, 18, 8, 6 and 16 (in decreasing order). By contrast, so-called “weak” clusters, with more than 50% of cases allocated with low probability (< 50%), were 10 and 19.

## Discussion

Following a brief training in the use of the MHCT, more than 12,000 people with severe mental disorders from the caseload of 53 CMHCs were assessed by means of the MHCT in a three months period. Professionals involved into the MHCT administration belonged to several categories, representing the multidisciplinary staff working in Italian mental health services. Of the total of trained professionals, three quarters used the MHCT, confirming that the training was useful to have the professionals able to administer it in routine practice without additional resources. Incomplete or inconsistent assessments were a small minority, again confirming that the professionals got a sufficient familiarity with the MHCT. However, some of the trained professionals had some previous experience with the HoNOS.

The allocation of the cases according to the algorithm translating the scores into the clusters was made centrally and automatically. This made the attribution to clusters easy and fast. In the UK, the attribution of the MHCT to clusters made directly by the professionals proved to be rather critical. We have shown that an automated attribution was feasible and that it was acceptable by the professionals, as witnessed by the low burden of the clustering process reported by the professionals.

Nonetheless, there are several pitfalls, practical and theoretical, in the assessment by means of the MHCT in the routine. The low degree of the certainty in the attribution to clusters for about one fourth of the cases encompasses that those people should have been reassessed. Since this was a pilot study, we did not indicate to reassess such cases, because it would have added some burden to the task. Anyway, the low probability in attribution to clusters concerned just a few clusters , and certainty was higher in the most prevalent clusters and lower in the small ones: 9% of cases were allocated to clusters with a degree of certainty of less than 50%.

All clusters included all the diagnoses considered and, viceversa, all the diagnoses included the most prevalent clusters. In clusters of low complexity also cases with diagnosis of schizophrenia were included, and in each diagnosis clusters of different severity were represented. This confirms that the clusters are relatively independent from the diagnoses, and they describe needs, problems, abilities, and environmental conditions of the individuals beyond the definition of the disorder.

In the cases assessed the most prevalent cluster was that describing ongoing recurrent psychosis with low symptomatology, followed by that including cases with non-psychotic disorders of moderate severity. This is not surprising and can correspond to a population of long-term users of psychiatric services in maintenance treatment. We were more surprised for the very low prevalence of first episode psychosis (cluster 10). However, a number of epidemiological studies of the caseload of Italian psychiatric services previously showed low figures of first onset psychoses, suggesting barriers to access of young people (Lora et al., 2012).

The comparison between the cluster distribution according to the level of complexity in this study and the distribution in English samples is difficult because our sample was selected by including just four diagnostic groups and therefore cannot be considered representative of the whole population treated by public psychiatric services. Moreover, the patients were assessed only once, and reassessment was not possible in cases of uncertain assignment. However, data of all patients admitted to mental health care in UK each year show that the cluster distribution was even more clear-cut, with the highest frequency of low complexity clusters and viceversa (Moscelli et al., 2019). In our sample, although the trend is the same, the clusters are more equally distributed.

Some limitations of this study should be acknowledged. The study was conducted on a subsample of cases defined according to four diagnostic groups and therefore did not represent the whole array of problems treated in the mental health services. The modifications introduced in the MHCT enhanced its suitability for use but limited the possibility of comparing the results with the available data on MHCT use in mental health care. The interrater reliability of cluster assignment was not evaluated. Last, but not least, the cross-sectional nature of this study prevented the longitudinal assessment of the correlation between the clusters, the resources use and the outcome of care, as well as the usefulness of MHCT as a basis for planning and evaluation.

## Conclusions

This was the first study on MHCT conducted in Europe outside UK. First and foremost, we showed that the use of a need assessment tool such as the MHCT was feasible by mental health professionals of various disciplines in routine practice of Italian psychiatric services following a brief training. The MHCT confirmed its properties to capture a variety of problems of people with mental disorders beyond the traditional psychiatric assessment, therefore giving the clinicians and patient themselves the opportunities of looking at services responses from a different viewpoint. The limitations of this study must be considered in relation to the main objective of providing a tool suitable in real world practice. However, this should be considered as a first step of a complex project aimed at studying the possibility of changes in funding and resource allocations to mental health services as a consequence of a thorough assessment of patients’ needs and the provision of tailored effective care. To what extent the information gathered through the MHCT can be used to provide a sound base for care planning and outcome evaluation remains to be seen. Some recent comments raised doubts about this possibility (Jacobs et al., 2019). Further developments of this project will address this issue.

## Data Availability

The data that support the findings of this study are available from the regions of Lombardy, Lazio, and Emilia-Romagna, and the Province of Palermo, but restrictions apply on the availability of these data, which were used under license for the current study, and so are not publicly available. Data are however available from the authors upon reasonable request and with permission from the regions involved in this study.

## List of Abbreviations

CMHCs: Community Mental Health Centers
BPD: Borderline Personality Disorder
DMHs: Departments of Mental Health
NHS: National Health Service
HoNOS: Health of the Nation Outcomes Scales
SARN: Summary Assessment of Risk and Need
HCU: Healthcare Utilization databases
GHPWs: General Hospital Psychiatric Wards
DCs: Day-Care Centers
CRFs: Community Residential Facilities
MHIS: Mental Health Information System
ICD: International Classification of Diseases
DSM: Diagnostic and Statistical Manual of mental disorders
ATC: Anatomical Therapeutic Chemical Classification System
SAS: Statistical Analysis System software
MoH: Ministry of Health
QUADIM: “Clinical pathways in patients with severe mental disorders in Italy” project
SMR: Standardized Mortality Ratio
ANOVA: Analysis of Variance
MHCT: Mental Health Clustering Tool
